# Gut microbiota–derived metabolites in immunomodulation and gastrointestinal cancer immunotherapy

**DOI:** 10.3389/fimmu.2025.1710880

**Published:** 2025-11-21

**Authors:** Wenbin Luo, Ruoyun Li, Chaofan Pan, Changjiang Luo

**Affiliations:** 1 The Second Clinical Medical School, Lanzhou University, Lanzhou, China; 2 Department of General Surgery, The Second Hospital of Lanzhou University, Lanzhou, China

**Keywords:** short-chain fatty acids (SCFAs), bile acids, tryptophan derivatives, tumor immune microenvironment (TIME), immune checkpoint inhibitors (ICBs), epigenetic, metabolic reprogramming

## Abstract

Gut microbiota-derived metabolites have emerged as critical mediators linking microbial composition with immune regulation and tumor progression in gastrointestinal (GI) cancers. This review highlights four major classes of metabolites: short-chain fatty acids (SCFAs), bile acids, tryptophan derivatives, and several emerging metabolites such as inosine, trimethylamine-N-oxide (TMAO), and urolithin A. These metabolites exert context-dependent effects by modulating innate and adaptive immune cells, shaping the tumor microenvironment, and influencing inflammatory and epigenetic pathways. Importantly, their immunoregulatory effects are not confined to the intestinal milieu but can extend systemically through both cellular and chemical routes. SCFAs and urolithin A enhance epithelial barrier integrity and CD8^+^ T cell activity but may also promote immune tolerance. Bile acids display dual roles, with ursodeoxycholic acid and tauroursodeoxycholic acid counteracting the tumor-promoting effects of deoxycholic acid and lithocholic acid. Tryptophan metabolism produces both immunosuppressive (kynurenine) and immune-protective (indole derivatives) metabolites that regulate T-cell differentiation and function. In addition, dietary interventions, probiotics, engineered microbes, and plant-derived nanoparticles offer novel strategies to reshape the microbiota–metabolite–immune axis and improve immunotherapy outcomes. To pinpoint the sites of metabolite action and mitigate translational risks, we highlight immune-competent organoid co-culture systems. These platforms enable quantitative assessment of exposure–response thresholds, dissection of context-dependent effects, and *in vitro* pre-evaluation of the feasibility and safety of metabolite-based immunologic adjuvants combined with PD-1/PD-L1 blockade. Collectively, microbiota-derived metabolites represent promising targets for precision diagnosis and treatment in GI cancer immunotherapy.

## 1 Introduction

Gastrointestinal (GI) cancers, including colorectal, gastric, hepatocellular, and biliary tract malignancies, remain among the leading causes of cancer-related morbidity and mortality worldwide (1, 2). In recent years, immune checkpoint inhibitors (ICIs), particularly antibodies targeting the PD-1/PD-L1 and CTLA-4 pathways, have revolutionized cancer therapy and significantly improved outcomes in subsets of patients (3, 4). However, their clinical efficacy is still limited by heterogeneous response rates, immune-related adverse events, and the development of primary or acquired resistance (5, 6). These challenges highlight the urgent need to identify novel biomarkers and adjuvant strategies to optimize immunotherapy efficacy in GI cancers.

Emerging evidence suggests that the gut microbiota and its metabolites play a pivotal role in shaping host immunity and influencing responses to ICIs (7). Microbial-derived metabolites, including short-chain fatty acids (SCFAs), bile acids, indole derivatives, and purine metabolites, can profoundly modulate the tumor immune microenvironment through multiple mechanisms, such as metabolic reprogramming, epigenetic regulation, and immune cell differentiation (8, 9). These metabolites exert context-dependent effects: they may enhance antigen presentation and cytotoxic T cell responses to strengthen antitumor immunity, or conversely promote immunosuppressive networks that facilitate tumor progression and therapeutic resistance.

In addition to mechanistic insights, clinical and translational studies have demonstrated that specific microbial taxa and their metabolic products are associated with improved ICI responsiveness and survival outcomes (10). Moreover, dietary interventions, probiotics, natural products, and engineered microbial therapies provide promising approaches to modulate the microbiome–metabolite–immune axis (11). These findings collectively underscore the importance of microbiota metabolites as therapeutic targets for cancer immunotherapy.

This review summarizes current insights into gut microbiota-derived metabolites in immune regulation and GI cancer immunotherapy, focusing on major metabolite classes, underlying mechanisms, clinical relevance, and prospects for metabolite-based strategies to enhance therapeutic efficacy and precision.

## 2 Gut microbiota–derived metabolites

### 2.1 Overview of gut microbiota–derived metabolites

Gut microbiota derived metabolites constitute a diverse group of low molecular weight compounds, primarily including SCFAs, bile acid derivatives, and tryptophan derivatives, as well as emerging molecules such as inosine, trimethylamine N oxide (TMAO), and urolithin A (UroA). Acting as key mediators of metabolic reprogramming, epigenetic regulation, and immune cell modulation, these metabolites collectively shape the tumor microenvironment and influence responses to immunotherapy. **Table 1** summarizes the major classes, microbial sources, metabolic pathways, and immunoregulatory functions of gut microbiota derived metabolites in GI cancers.

**Table 1:** Major classes, sources, and immunoregulatory functions of gut microbiota–derived metabolites in GI cancers.

Class of metabolite	Representative compounds	Major microbial sources	Biosynthetic/metabolic pathway	Key signaling targets/receptors	Major immunoregulatory or antitumor effects	Refs.
Short-chain fatty acids	Acetate, Propionate, Butyrate	*Clostridium*, *Faecalibacterium*, *Bacteroides*	Fermentation of dietary fibers, resistant starches, and oligosaccharides	GPR41/43, GPR109A, HDAC inhibition	Enhance epithelial barrier integrity; promote IL-10–STAT3 axis; regulate M1/M2 balance; improve CD8^+^ T-cell effector function; synergize with PD-1 therapy	(12–27)
Bile acids	CDCA, DCA, LCA, UDCA, TUDCA	*Clostridium*, *Eubacterium*, *Bacteroides*	Cholesterol catabolism and microbial dehydroxylation/deconjugation	FXR, TGR5, PXR	Regulate macrophage and T-cell polarization; suppress NF-κB activation; modulate ROS, m^6^A, and Treg stability; maintain epithelial homeostasis	(28–45)
Tryptophan metabolites	Kynurenine, IAA, IPA, IAld, 5-HT	*Lactobacillus*, *Bifidobacterium*, *Clostridium*	Kynurenine, indole, and serotonin pathways	AhR, PXR, IDO/TDO	Control Th17/Treg balance; induce IL-22; reinforce barrier integrity; modulate CD8^+^ T-cell infiltration; influence immune tolerance	(46–63)
Purine metabolites	Inosine, Guanosine	*Bifidobacterium*, *Faecalibaculum*	Purine degradation and salvage pathways	A2AR, cAMP–PKA	Enhance CD8^+^ activation; synergize with PD-1/CTLA-4 blockade; excessive adenosine promotes immunosuppression	(64, 65)
Trimethylamine-N-oxide (TMAO)	TMAO	*Lachnoclostridium*, *Desulfovibrio*	Oxidation of choline and carnitine	PERK–ER stress, IFN-I	Activate NLRP3 inflammasome; promote IFN-γ^+^TNF-α^+^ T-cell responses; dual effects on angiogenesis and immunity	(66–68)
Urolithin A (UroA)	UroA	*Gordonibacter*, *Ellagibacter*	Microbial conversion of ellagitannins/polyphenols	ERK1/2, ULK1	Improve CD8^+^ mitochondrial metabolism; enhance NK and CAR-T cytotoxicity; potentiate PD-1 blockade	(69, 70)
Formate	Formate	*Bacteroides*, *Clostridium*	Anaerobic fermentation and one-carbon metabolism	Nrf2	Induce Nrf2 activation and IFN-γ release; enhance antitumor T-cell function; link exercise and microbiota-mediated immunity	(71, 72)
L-Arginine/Arginine metabolism	Arginine, Ornithine, NO	*Bifidobacterium*, *Lactobacillus*	Urea cycle and arginase-dependent metabolism	mTOR, OXPHOS, FAO	Promote CD8^+^ memory differentiation; excessive arginase activity suppresses T-cell immunity	(73–75)
Vitamins (microbial B group)	B2, B5, B9, B12	Various commensal bacteria	Coenzyme synthesis/vitamin metabolism	MAIT activation, methylation enzymes	Support T-cell metabolism and Treg stability; enhance IL-22^+^ Tc22 differentiation	(73, 76)
Histidine-derived metabolite	UCA	*Muribaculum* spp.	Histidine degradation; induced by TKIs	IκBα, NF-κB–CXCL1 axis	Inhibits NF-κB–CXCL1 signaling in tumor endothelial cells; reduces MDSC infiltration; improves immune microenvironment; enhances anti-PD-1 efficacy	(77)
Phenylalanine-derived metabolite	4-HPA	Various gut bacteria	Shikimic-acid pathway; tyrosine/phenylalanine metabolism	JAK2/STAT3, CXCL3/CXCR2	Promotes CXCL3 expression and PMN-MDSC recruitment; suppresses CD8^+^ T-cell function; induces ICI resistance; inhibited by CGA	(78)
Selenium-containing amino acid	L-SeMet	Microbiota-associated selenium metabolism	Methionine and selenium assimilation pathway	LCK-TCR signaling	Enhances CD8^+^ T-cell IFN-γ, TNF-α, GzmB, IL-2 production; strengthens cytotoxicity and antitumor immunity	(79)

SCFAs, short-chain fatty acids; CDCA, chenodeoxycholic acid; DCA, deoxycholic acid; LCA, lithocholic acid; UDCA, ursodeoxycholic acid; TUDCA, tauroursodeoxycholic acid; FXR, farnesoid X receptor; TGR5, Takeda G protein–coupled receptor 5 (also known as GPBAR1); PXR, pregnane X receptor; AhR, aryl hydrocarbon receptor; IDO, indoleamine 2,3-dioxygenase; TDO, tryptophan 2,3-dioxygenase; Kyn, kynurenine; IAA, indole-3-acetic acid; IPA, indole-3-propionic acid; IAld, indole-3-aldehyde; 5-HT, 5-hydroxytryptamine (serotonin); HDAC, histone deacetylase; GPR, G protein–coupled receptor; GPR41/43, G protein–coupled receptor 41/43; GPR109A, G protein–coupled receptor 109A; NF-κB, nuclear factor kappa-light-chain-enhancer of activated B cells; IL, interleukin; ILC, innate lymphoid cell; Treg, regulatory T cell; Th17, T helper 17 cell; STAT3, signal transducer and activator of transcription 3; PD-1, programmed cell death protein 1; CTLA-4, cytotoxic T-lymphocyte antigen 4; A2AR, adenosine A2A receptor; cAMP, cyclic adenosine monophosphate; PKA, protein kinase A; ERK1/2, extracellular signal–regulated kinase 1/2; ULK1, unc-51-like autophagy activating kinase 1; Nrf2, nuclear factor erythroid 2–related factor 2; NLRP3, NOD-, LRR-, and pyrin domain-containing protein 3; IFN-γ, interferon gamma; TNF-α, tumor necrosis factor alpha; PERK, protein kinase RNA-like endoplasmic reticulum kinase; IFN-I, type I interferon; mTOR, mechanistic target of rapamycin; OXPHOS, oxidative phosphorylation; FAO, fatty acid oxidation; MAIT, mucosal-associated invariant T cell; ROS, reactive oxygen species; m^6^A, N6-methyladenosine; NO, nitric oxide; CAR-T, chimeric antigen receptor T cell. UCA, urocanic acid; TKI, tyrosine kinase inhibitor; MDSC, myeloid-derived suppressor cell; 4-HPA, 4-hydroxyphenylacetic acid; JAK2, Janus kinase 2; CXCL3, C-X-C motif chemokine ligand 3; CXCR2, C-X-C motif chemokine receptor 2; CGA, chlorogenic acid; L-SeMet, L-selenomethionine; LCK, lymphocyte-specific protein tyrosine kinase; TCR, T-cell receptor; GzmB, granzyme B.

### 2.2 Systemic distribution and immune crosstalk of microbiota-derived metabolites

The metabolic activity of the gut microbiota extends beyond the intestinal milieu and influences systemic immunity through two complementary routes: a chemical route and a cellular route. In the chemical route, small microbial metabolites are absorbed through the intestinal epithelium into the portal circulation (80), processed in the liver, and subsequently spill over into the systemic bloodstream (81, 82). In parallel, a portion of these molecules is carried by afferent lymph to the draining mesenteric lymph nodes (MLNs) (83, 84). This dual vascular–lymphatic conduit enables microbial metabolic signals to reach peripheral immune sites such as the liver, spleen, and MLNs. During development, maternally derived microbiota-dependent metabolites can traverse the placenta and modulate fetal immune organogenesis, thereby establishing a metabolic foundation for thymic maturation and immune tolerance (85, 86).

In the cellular route, dendritic cells and T cells conditioned by microbial metabolites egress via lymphatic vessels and home to the MLNs under the guidance of the C-C chemokine receptor 7 (CCR7)–C-C motif chemokine ligands 19 and 21 (CCL19/CCL21) axis (87, 88). Within these lymphoid niches, they prime naïve T cells and imprint tissue-homing programs (89). The resulting gut-conditioned effector and regulatory T cells subsequently disseminate through the circulation to peripheral organs, translating local metabolic cues into systemic immune effects (90). In adults, there is currently no conclusive evidence that gut-derived metabolites can cross the blood–thymus barrier and directly act on thymic tissue. This is likely related to the selective permeability of the cortical barrier region, which restricts molecular access under steady-state conditions (91). Instead, a more widely recognized mechanism involves cell-mediated communication, whereby dendritic cells acquire microbial or peripheral antigens from the circulation and deliver them to the thymus, enabling intestinal signals to indirectly influence thymic selection and the maintenance of immune homeostasis (92, 93).

Collectively, the diffusion of metabolites and trafficking of immune cells form a bidirectional communication axis through which microbial metabolic activity in the gut becomes systemically perceptible, fostering dynamic crosstalk and coordinated regulation between the intestinal and peripheral immune systems.

### 2.3 SCFAs

#### 2.3.1 Biosynthesis and metabolic functions

SCFAs, primarily including acetate, propionate, and butyrate, are produced by anaerobic gut microbiota through the fermentation of dietary fibers, resistant starch, and oligosaccharides (14). In the colonic lumen, their concentrations can reach 50–150 mM, serving as an important energy source for intestinal epithelial cells (15). Among them, butyrate is particularly critical for maintaining colonic barrier homeostasis, whereas acetate and propionate are more likely to enter the systemic circulation (16), where they participate in hepatic gluconeogenesis, lipid synthesis, and immunometabolism (17).

Beyond their local metabolic roles, SCFAs can also engage extra-intestinal immunity through distinct distribution routes. They reach peripheral sites primarily via a chemical route, beginning with epithelial uptake into the portal vein, followed by hepatic processing and partial spillover into the systemic circulation (94). In parallel, afferent lymph exposes the draining MLNs to these metabolites (95). When systemic exposure is limited (96), a cellular route also contributes (97), whereby SCFA-conditioned dendritic cells and T cells egress via the lymphatics and, guided by the CCR7–CCL19/CCL21 axis, home to MLNs before disseminating to peripheral tissues (88, 98).

#### 2.3.2 Mechanistic roles in cancer biology

The roles of SCFAs in GI cancers are primarily mediated through two major mechanisms. First, via metabolic regulation, SCFAs enter cells through monocarboxylate transporters and serve as metabolic substrates, thereby modulating energy metabolism and inflammatory responses (18). Second, through immune regulation, SCFAs activate G-protein–coupled receptor 43 (GPR43, also known as free fatty acid receptor 2, FFAR2) and G-protein–coupled receptor 109A (GPR109A, also known as hydroxycarboxylic acid receptor 2, HCAR2), and inhibit histone deacetylases (HDACs), which influence chromatin structure and transcription factor occupancy, ultimately regulating gene expression (19). Previous studies have demonstrated that these effects are essential for maintaining intestinal barrier function and homeostasis. For example, butyrate can preserve mucosal barrier integrity and suppress proinflammatory responses through its roles in energy provision and epigenetic regulation, while exhibiting antiproliferative and pro-apoptotic effects in colorectal cancer (20, 21). Beyond barrier regulation, SCFAs also exert direct effects on immune cell function. For example, butyrate enhances CD8^+^ T cell effector activity by upregulating inhibitor of DNA binding 2 (ID2) through HDACs inhibition, thereby markedly strengthening antitumor immunity in colorectal cancer models. Moreover, SCFAs—particularly butyrate and propionate—can modulate signaling pathways and transcriptional programs to downregulate the expression of vascular endothelial growth factor (VEGF) and neuropilin-1, thereby inducing autophagy, ultimately suppressing tumor angiogenesis and limiting the proliferation of colorectal cancer cells (22, 23). Notably, the butyrate-producing strain CBM588 has been shown in clinical trials to improve response rates and progression-free survival (PFS) when combined with ICIs, highlighting the translational potential of targeting SCFA metabolism (24). Clinical evidence also supports this notion, in patients with solid tumors receiving anti–PD-1 therapy, elevated fecal levels of acetate, propionate, butyrate, and valerate were significantly associated with prolonged PFS and improved therapeutic responses (25). In contrast, patients with hepatocellular carcinoma (HCC) exhibit a marked reduction of SCFA-producing bacteria, leading to decreased levels of butyrate and propionate, thereby promoting inflammation, fibrosis, and tumorigenesis (26, 27).

Nevertheless, the roles of SCFAs remain controversial. Some studies have reported that elevated levels of butyrate and propionate are associated with resistance to CTLA-4 blockade and an increased proportion of regulatory T cells (Tregs), suggesting that SCFAs may attenuate CTLA-4–induced antitumor immunity. Acetate, in certain contexts, can even promote tumor growth and immune evasion through the acetyl coenzyme A (acetyl-CoA)–cellular myelocytomatosis oncogene (c-Myc)–PD-L1 signaling pathway (12, 13). Building on this, recent studies have further revealed that SCFAs promote tumor growth and immune evasion through the interconnected processes of metabolic reprogramming, epigenetic modification, and immune regulation. Butyrate modulates cellular energy metabolism by inhibiting HDACs and activating AMP-activated protein kinase (AMPK), thereby indirectly reshaping N^6^-methyladenosine (m^6^A) modification patterns that contribute to immune evasion and metabolic reprogramming. In parallel, propionate alters histone acetylation and affects methyltransferase-like 3 (METTL3)–mediated m^6^A deposition, promoting tumor survival and therapy resistance through the regulation of lipid metabolism and ferroptosis tolerance (99). These m^6^A alterations exert profound effects on the fate of transcripts involved in immune regulation and metabolism. Enhanced METTL3-dependent methylation destabilizes suppressor of cytokine signaling 1 (SOCS1) and phosphatase and tensin homolog deleted on chromosome 10 (PTEN) mRNAs while stabilizing c-Myc and PD-L1, thereby sustaining oncogenic signaling and facilitating immune escape (100, 101). Inhibition of the demethylase fat mass and obesity-associated protein (FTO), potentially triggered by butyrate-induced AMPK activation, leads to increased m^6^A deposition on STAT1 and interferon-responsive transcripts, extending their mRNA half-life and amplifying antitumor immune activation (102). In regulatory T cells, the METTL3/METTL14–YT521-B homology domain family (YTHDF) axis preserves forkhead box protein P3 (FOXP3) expression and suppressive stability through selective m^6^A modification (103, 104). Collectively, SCFA-driven epitranscriptomic remodeling establishes a mechanistic bridge that links microbial metabolism to gene-expression dynamics and immune phenotypes, integrating metabolic, epigenetic, and immunologic signaling into a unified regulatory network (105).

#### 2.3.3 Immunoregulatory effects and immune-cell mechanisms

SCFAs profoundly influence both innate and adaptive immunity. In macrophages, butyrate acts through GPR109A/FFAR2 and HDACs inhibition to suppress nuclear factor kappa-light-chain-enhancer of activated B cells (NF-κB) signaling and activate the IL-10–STAT3 axis, thereby driving M2-like polarization (106). Under immune-active conditions, moderate butyrate signaling enhances macrophage antigen-presenting capacity and synergizes with CD8^+^ T-cell responses (107). SCFAs also promote dendritic-cell maturation and antigen presentation via GPR41/GPR43 signaling, although high concentrations of butyrate can suppress pro-inflammatory cytokine secretion (108). Furthermore, SCFAs enhance NK-cell cytotoxicity and IFN-γ secretion (109), and stimulate innate lymphoid cells (ILCs) activity by promoting IL-22 production through HDACs inhibition (110). At the mucosal barrier, they strengthen tight-junction proteins (Claudin-1, occluding, zonula occludens-1) and enhance Mucin 2 expression to restore epithelial-barrier integrity (111, 112). Consistently, in a mouse model of non-alcoholic fatty liver disease–associated hepatocellular carcinoma, *Bifidobacterium pseudolongum*–derived acetate restored microbial balance and improved barrier function (113). Within adaptive immunity, SCFAs induce Treg differentiation and IL-10 secretion but also improve CD8^+^ T-cell mitochondrial fitness (114), underscoring their context-dependent dual functions in immune tolerance and antitumor activation (115, 116).

**Figure 1:**
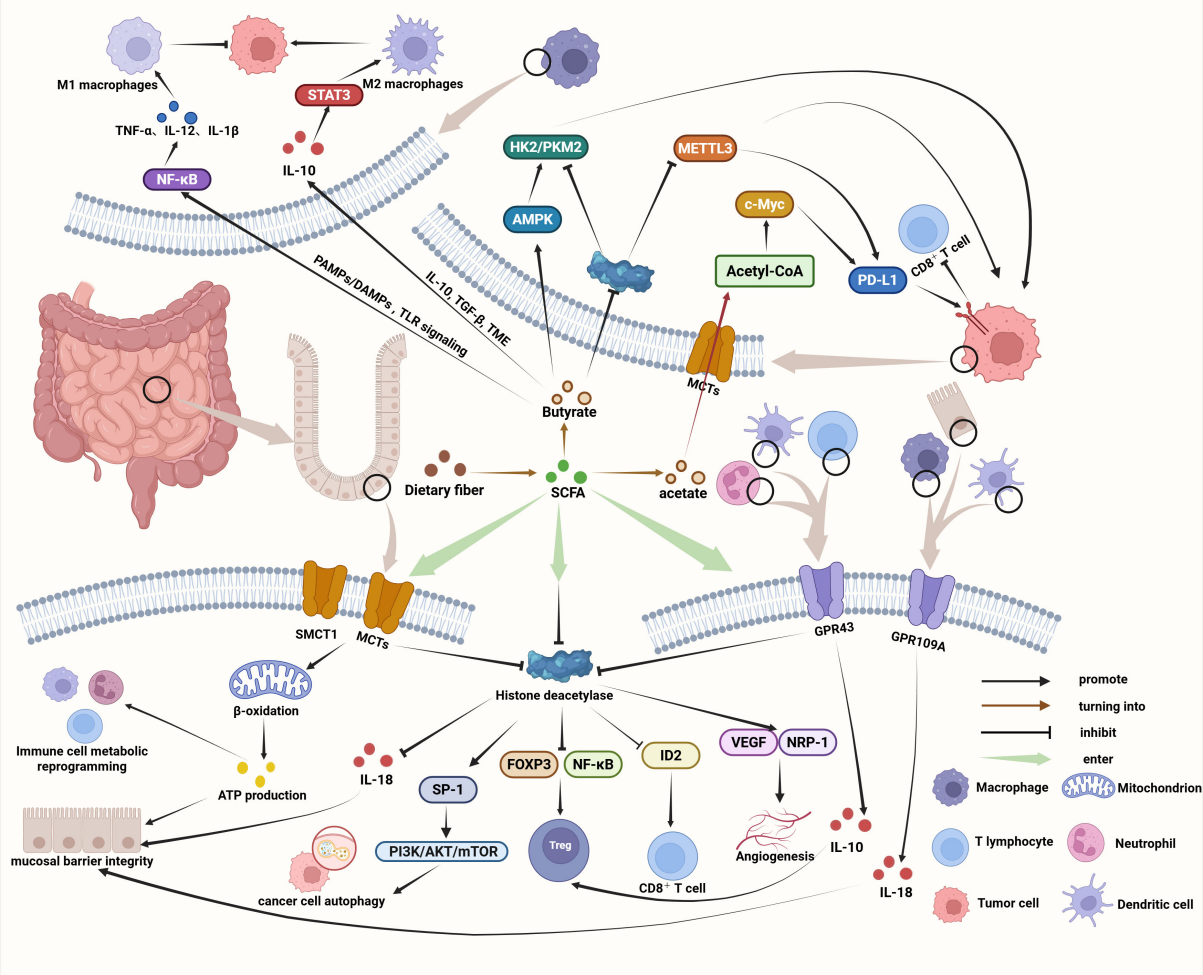
Mechanistic and immunoregulatory roles of SCFAs in GI cancers. Created in https://BioRender.com.

In GI cancers, SCFAs exert dual and context-dependent effects through the integrated regulation of metabolism, epigenetics, and immunity. On one hand, they provide energy, preserve epithelial-barrier integrity, and suppress tumor growth; on the other hand, under specific microenvironmental conditions, they may induce immune tolerance and impair immune surveillance. Their overall influence depends on factors such as metabolite concentration, microbiota composition, host immune status, and therapeutic context. Collectively, SCFAs orchestrate metabolic and immune networks that govern tumor progression and antitumor responses (**Figure 1**).

### 2.4 Bile acid derivatives

#### 2.4.1 Biogenesis and microbial transformation

Bile acids are important endogenous metabolites synthesized in the liver and further transformed by the gut microbiota. They not only participate in lipid digestion and absorption but also act as signaling molecules that play critical roles in metabolic regulation (29). Based on their origin, BAs can be classified into primary bile acids and secondary bile acids. Primary bile acids are synthesized in the liver from cholesterol, whereas secondary bile acids are generated in the intestine through bacterial reactions such as dehydroxylation, deconjugation, and oxidation–reduction (30).

In addition to their biochemical transformations, bile acids display unique distribution dynamics and immunoregulatory properties along the gut–liver axis. Primary bile acids and their microbially derived metabolites circulate predominantly via enterohepatic circulation, with limited spillover into the systemic circulation (117), establishing endocrine-like exposure along the gut–liver–lymphoid axis (118). In parallel, MLNs receive additional inputs via afferent lymphatics (119). Meanwhile, secondary bile acids locally condition myeloid and lymphoid immune cells in the intestine and liver, and the resulting gut-imprinted immune programs disseminate system-wide via the cellular route of immune-cell trafficking (120, 121).

#### 2.4.2 Dual roles in tumorigenesis and immune modulation

Bile acid signaling is predominantly mediated through the nuclear receptor farnesoid X receptor (FXR) and the membrane-bound G protein–coupled receptor TGR5 (GPBAR1), which orchestrate the regulation of bile acid synthesis, lipid metabolism, energy homeostasis, and inflammatory responses (30, 31). In GI cancers, these signaling pathways are closely linked to mucosal barrier homeostasis, the inflammatory microenvironment, and immune regulation. The primary bile acid chenodeoxycholic acid (CDCA) can activate TGR5, promoting the secretion of anti-inflammatory factors and suppressing proinflammatory signaling, thereby helping to maintain mucosal homeostasis and immune tolerance. This effect may exert a protective role in inflammation-driven tumorigenesis (8, 32). Notably, in hepatocellular carcinoma models, primary bile acids can upregulate C-X-C motif chemokine ligand 16 (CXCL16) expression on liver sinusoidal endothelial cells (LSECs), thereby recruiting and activating Natural Killer T (NKT) cells to enhance antitumor immunity. In contrast, secondary bile acids downregulate CXCL16 expression and reduce NKT cell infiltration, thereby attenuating antitumor effects (33). Clinically, gene expression profiling of patients with HCC has shown that high expression of bile acid metabolism–related genes is positively correlated with increased CD8^+^ T cell infiltration and improved prognosis (34). A multi-omics study further confirmed that patient subgroups with high bile acid metabolic activity exhibit stronger immune cell infiltration and better survival outcomes, whereas low metabolic activity is associated with immunosuppressive phenotypes and poor prognosis (35). Another metabolomics analysis revealed a significant enrichment of secondary bile acid synthesis pathways in HCC patients, suggesting that secondary bile acid metabolism may represent a key driver of HCC progression and clinical outcome (36).

High concentrations of secondary bile acids such as deoxycholic acid (DCA) can promote colorectal carcinogenesis by inducing oxidative stress, DNA damage, and proinflammatory signaling (37). Specifically, DCA drives reactive oxygen species (ROS) production and activates activator protein-1 (AP-1), NF-κB, and cyclooxygenase-2 (COX-2) pathways in colonic epithelial cells, while in cancer cells it further induces DNA damage and mutation accumulation, thereby accelerating tumor progression (38). In addition, DCA can activate the epidermal growth factor receptor (EGFR)/phosphoinositide 3-kinase (PI3K)/protein kinase B (AKT)/mechanistic target of rapamycin (mTOR) signaling axis by inducing Epidermal Growth Factor (EGF) release and membrane structural damage, thereby promoting cell proliferation and epithelial–mesenchymal transition (EMT), ultimately driving tumor invasion, metastasis, and immune evasion (39). Recent studies have revealed that gut-derived secondary bile acids can synergize with lipoteichoic acid (LTA), a product of Gram-positive bacteria, to activate toll-like receptor 2 (TLR2) signaling, thereby inducing hepatic stellate cells to upregulate COX-2 expression and increase Prostaglandin E2 production, which in turn shapes an immunosuppressive microenvironment and attenuates antitumor immunity (40). At the epigenetic level, DCA can bind to and inhibit the m^6^A demethylase FTO, thereby increasing m^6^A modification of transcripts such as FOXP3 and CTLA-4, which enhances Treg stability and immunosuppressive activity. Conversely, lithocholic acid (LCA) downregulates ALKBH5 through the pregnane X receptor (PXR) pathway, resulting in elevated m^6^A deposition on Wnt/β-catenin target transcripts and consequently promoting cancer stemness and therapeutic resistance (99). Functionally, m^6^A enrichment on FOXP3 and CTLA-4 mRNAs improves their stability and translation efficiency, reinforcing Treg differentiation and suppressive potential (122). Similarly, increased m^6^A modification on Wnt pathway effectors such as MYC and CCND1 strengthens β-catenin signaling and drives tumor stemness, thereby fostering resistance to therapy (123, 124). Emerging evidence further indicates that secondary bile acids like DCA not only inhibit FTO but also disrupt the METTL3–METTL14–Wilms tumor 1–associating protein (WTAP) complex, leading to global m^6^A accumulation, whereas LCA suppresses AlkB homolog 5 (ALKBH5) via PXR signaling to amplify Wnt/β-catenin activation (125, 126). In addition, ALKBH5 has been identified as a pivotal regulator of PD-L1 expression and immune microenvironment remodeling in intrahepatic cholangiocarcinoma (127). Collectively, these findings demonstrate that secondary bile acids remodel the epitranscriptome to concurrently stabilize Treg-mediated immune tolerance and sustain oncogenic signaling. Integration of bile-acid metabolism with RNA methylation thus delineates a coherent mechanistic continuum from metabolic dysregulation to immune escape and therapeutic resistance (128).

In contrast, ursodeoxycholic acid (UDCA) exerts antagonistic effects: it not only downregulates EGFR expression and suppresses EMT (41, 42), but also inhibits tumor metastasis by enhancing epithelial cadherin expression. Its antitumor efficacy is comparable to that of the EGFR inhibitor gefitinib, and their combined application can even produce synergistic effects (43). Moreover, a decrease in the protective bile acid tauroursodeoxycholic acid (TUDCA) weakens anti-inflammatory and barrier-protective functions, while secondary bile acids have been shown to directly promote cholangiocarcinoma through ROS generation and DNA damage (37, 44). Notably, secondary bile acids also play a crucial role in tumor metastasis. Li et al. reported that LCA activates TGR5 signaling to upregulate CCL3/CCL4 secretion by cancer-associated fibroblasts (CAFs), thereby recruiting myeloid-derived suppressor cells (MDSCs) into hepatic metastatic niches and significantly promoting the progression of colorectal cancer liver metastasis (CRLM) (45).

#### 2.4.3 Immune-cell regulation by bile-acid metabolites

Secondary bile acids such as DCA and LCA favor M2 macrophage polarization and foster an immunosuppressive microenvironment. Conversely, protective bile acids (TCDCA, UDCA) attenuate chronic inflammation by inhibiting NF-κB through the FXR/TGR5 axis (28). Bile-acid signaling also regulates dendritic-cell differentiation via FXR/TGR5 (82) and suppresses ILC3 function, weakening barrier defense (129). At the adaptive level, iso-deoxycholic acid (isoLCA) antagonizes RORγt to suppress Th17 differentiation and expand Tregs (8, 130), while DCA induces ROS and ER stress to impair CD8^+^ T-cell function (131, 132). These findings demonstrate that bile acids coordinate macrophage, DC, ILC, and T-cell functions to balance immune activation and tolerance in the tumor microenvironment.

**Figure 2:**
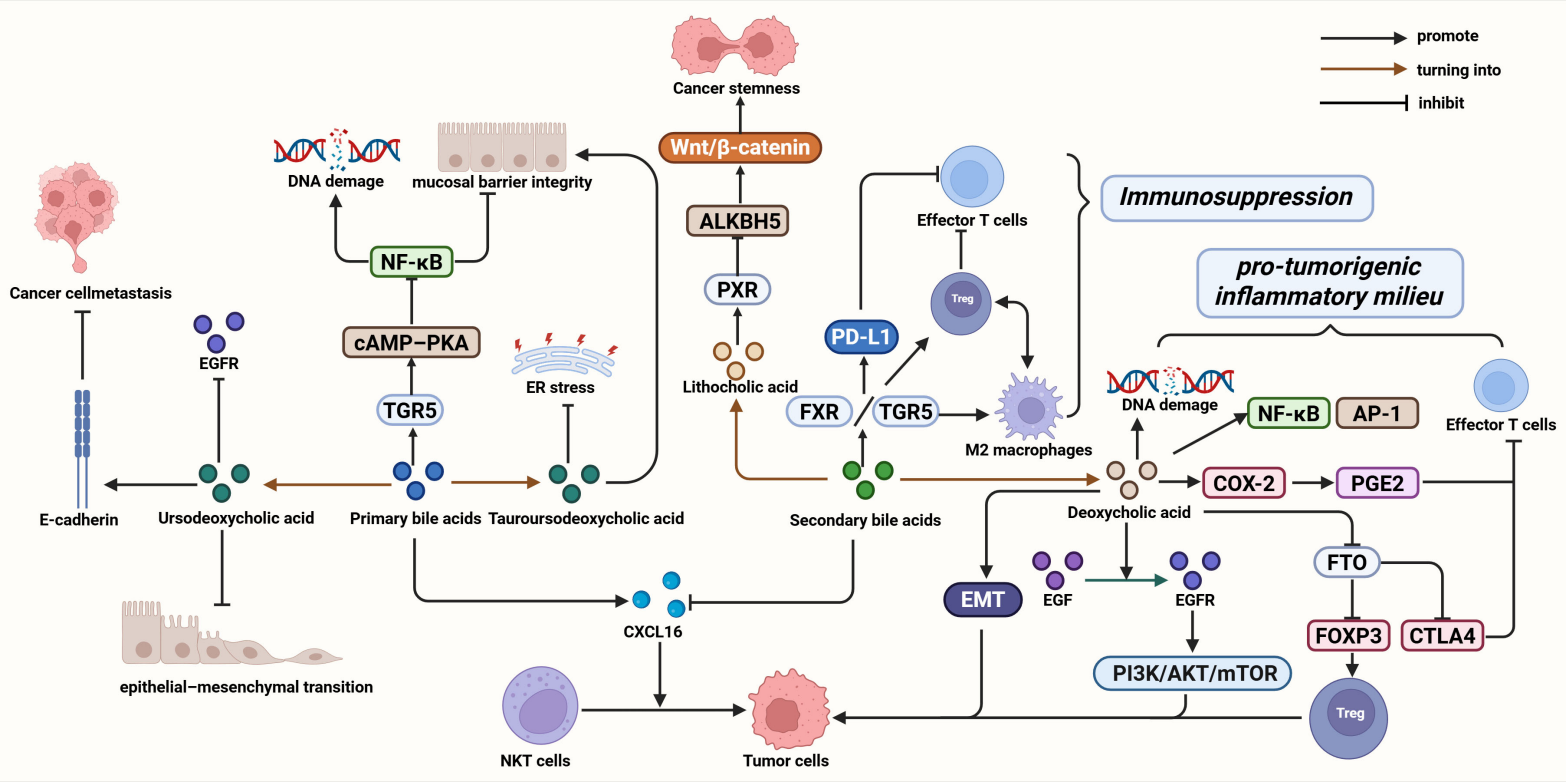
Dual roles and immunoregulatory mechanisms of bile-acid metabolites in GI cancers. Created in https://BioRender.com.

In GI cancers, bile acids act as bidirectional regulators of tumor biology and immune homeostasis. Activation of FXR and TGR5 signaling by primary and conjugated bile acids preserves epithelial integrity, suppresses inflammation, and promotes cytotoxic lymphocyte recruitment, thereby supporting antitumor immunity. In contrast, excessive secondary bile acids induce oxidative stress, DNA damage, and immunosuppressive signaling through ROS generation, m^6^A modification, and Treg stabilization. By integrating metabolic signaling with receptor-mediated immune regulation, bile acids fine-tune the balance between tumor promotion and immune surveillance, ultimately influencing disease progression and therapeutic responsiveness (**Figure 2**).

### 2.5 Tryptophan metabolites

#### 2.5.1 Metabolic pathways and major derivatives

Tryptophan (Trp) is an essential amino acid in humans, and its metabolism proceeds through multiple pathways under the coordinated action of the host and gut microbiota, primarily including the kynurenine pathway, the indole pathway, and the serotonin (5-HT) pathway (49). These metabolic routes not only contribute to protein synthesis, 5-HT production, and energy metabolism but also play critical roles in regulating immune responses and maintaining intestinal homeostasis through their metabolites. Emerging evidence further suggests that tryptophan-derived metabolites hold potential as therapeutic targets in cancer immunotherapy (50).

Functionally, tryptophan-derived metabolites influence systemic immunity through both chemical and cellular routes. Circulating kynurenine and 5-HT deliver direct chemical-route cues to peripheral immune and stromal cells (133, 134). By contrast, microbiota-derived indoles act predominantly locally, programming epithelial and antigen-presenting cells (135, 136). The resulting imprinted dendritic cells and T cells then traffic via the lymphatics to MLNs and disseminate through the cellular route, extending indole-driven effects to distant tissues (137, 138).

#### 2.5.2 Roles in tumorigenesis and immune homeostasis

In the kynurenine pathway, approximately 90–95% of Trp is converted into kynurenine (Kyn) by indoleamine 2,3-dioxygenase (IDO) or tryptophan 2,3-dioxygenase (TDO), and its downstream metabolites are extensively involved in energy metabolism and immune regulation. In GI cancers, IDO1 is often highly expressed, leading to elevated Kyn levels, which not only reflect metabolic reprogramming of tumor cells but also contribute to the formation of an immunosuppressive microenvironment (51).

In the indole pathway, about 5% of Trp is metabolized by the gut microbiota into various indole derivatives, including indole-3-acetic acid (IAA), indole-3-propionic acid (IPA), indole-3-aldehyde (IAld), and skatole. These metabolites act primarily through the aryl hydrocarbon receptor (AhR) and the PXR (52), and in GI cancers they may exert protective effects—for example, IAA and IPA can strengthen the epithelial barrier and suppress inflammation, thereby reducing cancer risk (53). However, in patients with HCC, IPA levels are markedly reduced, weakening epithelial barrier function and facilitating pathogen translocation and inflammation, thus promoting tumorigenesis (54). Certain indole metabolites may also drive proinflammatory or immunosuppressive responses under specific conditions, such as skatole, which induces inflammatory cytokine release, or IAA, which in some tumors activates AhR to promote immune evasion and suppress antitumor immunity (55).

In the 5-HT pathway, a small fraction of Trp is converted into serotonin by tryptophan hydroxylase 1 (TPH1), primarily regulating gut motility, vascular function, and neuroimmune interactions (56). Studies in GI cancers have shown that 5-HT is associated with intestinal epithelial proliferation and local immune status, and its metabolic imbalance may contribute to the remodeling of the tumor microenvironment (50, 57).

Tryptophan metabolites constitute a host–microbiota metabolic network in GI cancers, mediating a balance between barrier protection and anti-inflammatory effects on one side, and immunosuppression and tumor progression on the other, thereby serving as critical links connecting diet, microbiota, metabolism, and tumor immunity.

#### 2.5.3 Immunomodulatory mechanisms of tryptophan-derived metabolites

**Figure 3:**
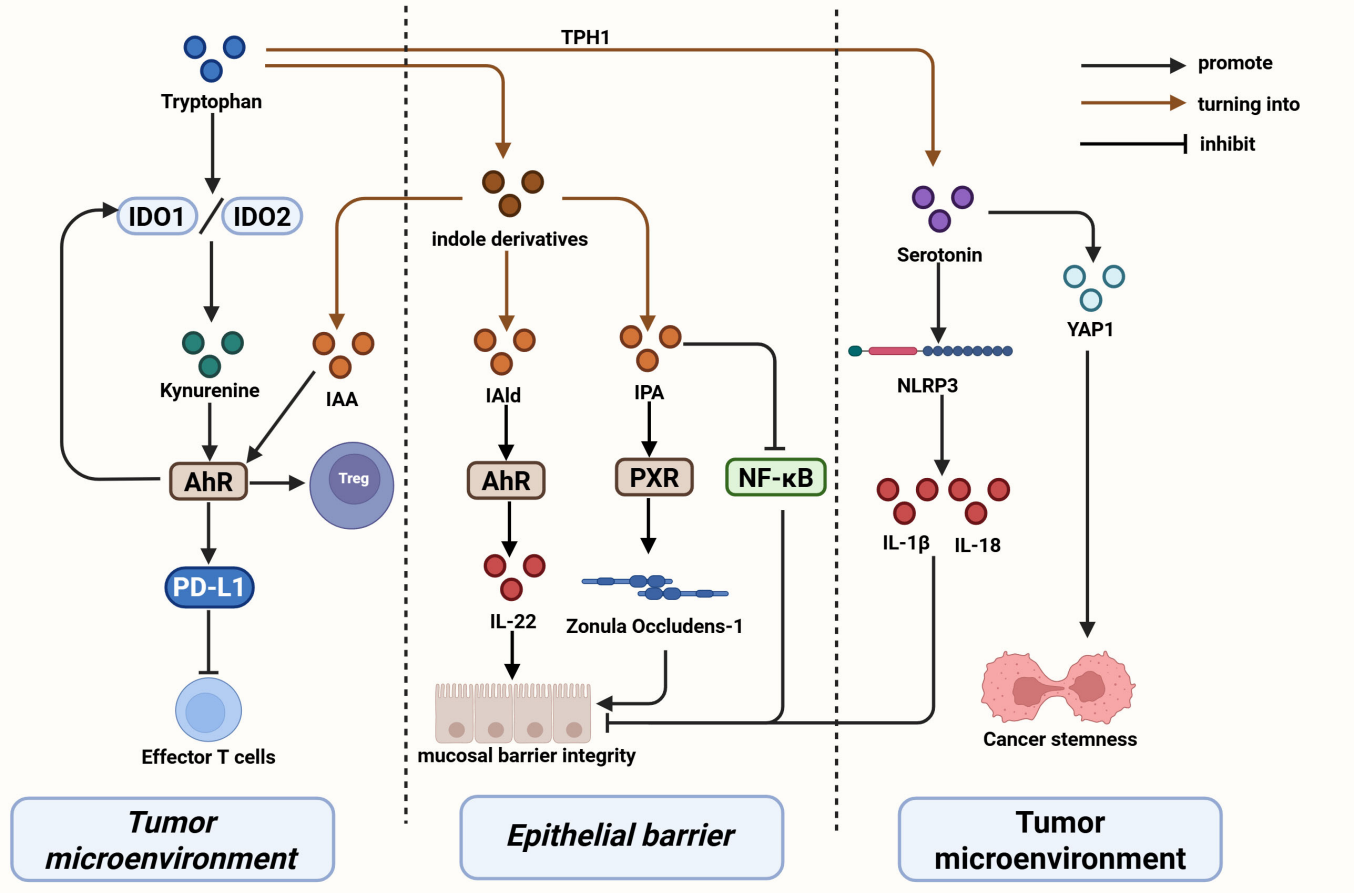
Metabolic and immunoregulatory mechanisms of tryptophan-derived metabolites in GI cancers. Created in https://BioRender.com.

Indole derivatives such as IAA and IPA exert anti-inflammatory functions via AhR signaling, limiting M1 macrophage polarization and maintaining mucosal homeostasis (58, 59). They also induce IL-22 production by ILC3s and enhance barrier integrity (60, 61). In contrast, kynurenine accumulation through IDO1 overexpression activates AhR to upregulate Siglec-15, suppress CD8^+^ T-cell infiltration, and promote immune evasion (62, 63). IAA and IPA reinforce the Th17/Treg balance, increase IL-10 and IFN-γ production, and stimulate CD4^+^/CD8^+^ T-cell activation (46–48). Overall, tryptophan metabolites act at multiple levels, from macrophages and ILCs to adaptive lymphocytes, to orchestrate immune homeostasis and antitumor responses. The major metabolic and immunoregulatory mechanisms of tryptophan-derived metabolites are summarized in **Figure 3**.

### 2.6 Other emerging metabolites

In addition to SCFAs, bile acids, and tryptophan derivatives, recent metabolomic and functional studies have identified a variety of emerging microbiota-derived metabolites (114). These molecules not only participate in maintaining host metabolic homeostasis but also modulate immune cell function and reshape the tumor microenvironment, thereby exerting significant and complex effects on GI tumorigenesis and responses to immunotherapy (11).

The underlying mechanisms by which these metabolites exert their systemic and immunological effects involve both chemical and cellular routes. One class of molecules such as inosine, TMAO and UroA can be detected in peripheral circulation (139–141), indicating their systemic exposure via intestinal absorption, portal venous transport, hepatic processing, and recirculation through the bloodstream and lymphatics (142). These metabolites have been reported to modulate immune microenvironments across organs and to influence antitumor immune responses (143). Other emerging metabolites primarily condition intestinal immune cells locally; these gut-imprinted cells then migrate through MLNs to disseminate their effects systemically via a cellular route (83, 144). In addition, vitamin-like or small molecules that are highly sensitive to dietary or therapeutic interventions show markedly context-dependent levels of peripheral exposure and immunological outcomes (145–147).

#### 2.6.1 Overview and biological significance

Inosine, a key intermediate of purine metabolism produced by both host and microbiota, enhances effector T cell activity via the adenosine A2A receptor (A2AR)–cyclic adenosine monophosphate (cAMP)–protein kinase A (PKA) axis and synergizes with PD-1/CTLA-4 blockade, showing translational potential in colorectal cancer (65). However, purine metabolism exerts complex effects, while AMPK/mTOR signaling and abnormal accumulation of metabolites such as hypoxanthine and guanosine can promote inflammation, oxidative stress, and tumor survival, enzymes in purine biosynthesis have emerged as therapeutic targets to reverse immunosuppression (64). Thus, purine metabolites may function either as immunotherapy sensitizers or as drivers of immune evasion, highlighting the need for context-specific understanding and intervention.

TMAO, derived from microbial metabolism of choline and carnitine, has recently attracted attention for its immunological functions. In colorectal cancer models, TMAO activates type I interferon signaling to enhance immune infiltration and improve chemo- and immunotherapy efficacy, while clinical studies link elevated TMA levels to survival benefit (67). Mechanistically, TMAO can act as an adjuvant to ICIs by inducing PKR-like endoplasmic reticulum kinase (PERK)–mediated endoplasmic reticulum stress and gasdermin E (GSDME)–dependent pyroptosis, thereby boosting CD8^+^ T cell antitumor activity (68). However, TMAO may also promote VEGF secretion and angiogenesis in colorectal cancer cells, suggesting dual roles in immunostimulation and tumor progression, with context-dependent outcomes (66).

UroA, a metabolite derived from dietary polyphenols such as ellagic acid and ellagitannins through gut microbiota metabolism, can improve mitochondrial function, suppress chronic inflammation, and modulate gut microbial metabolism. These effects suggest that UroA holds significant value in the prevention of GI cancers and the regulation of antitumor immunity (70). Recent studies further show that UroA activates extracellular signal–regulated kinase 1/2 (ERK1/2) to enhance CD8^+^ T cell effector function, persistence, and CAR-T cell metabolic fitness (69), suggesting its dual potential as a protective factor and immunotherapy sensitizer in GI cancers.

L-Arginine (L-arginine, Arg) is an important amino acid metabolite that functions as a key intermediate in the urea cycle and a precursor of nitric oxide (NO). In addition to its roles in nitrogen metabolism and vascular homeostasis, Arg also regulates immune cell function. Studies have shown that Arg supplementation shifts the metabolism of activated T cells from glycolysis toward oxidative phosphorylation (OXPHOS) and fatty acid oxidation (FAO), thereby promoting the differentiation and long-term survival of central memory–like T cells, enhancing CD8^+^ T cell cytotoxicity, and improving the efficacy of immune checkpoint blockade (ICB) (75). Conversely, within the GI tumor microenvironment, myeloid-derived suppressor cells (MDSCs) and TAMs frequently overexpress arginases (74), accelerating Arg depletion and leading to local immunosuppression and T cell dysfunction (73). A fasting-mimicking diet enriches Bifidobacterium pseudolongum, increases Arg accumulation, and promotes CD8^+^ tissue-resident memory T cell differentiation, thereby enhancing anti-CTLA-4 efficacy. Targeting Arg metabolism through supplementation or arginase inhibition, combined with dietary and microbiota modulation, offers a promising strategy to improve the tumor immune microenvironment and immunotherapy sensitivity in GI cancers (148).

Microbiota-derived vitamins, particularly B vitamins, are increasingly recognized in GI cancer immunity. Riboflavin (B2) metabolites activate mucosal-associated invariant T (MAIT) cells, folate (B9) supports Treg stability and DNA methylation, and cobalamin (B12) is linked to energy metabolism and colorectal cancer risk. Notably, pantothenic acid (B5), as a precursor of coenzyme A, promotes CD8^+^ T cells to differentiate into IL-22^+^ Tc22 cells (73), enhancing antitumor immunity and immunotherapy efficacy. Both preclinical and clinical studies suggest that specific B vitamins may serve as adjuvants and predictive biomarkers for immunotherapy (76).

Formate, a key intermediate of one-carbon metabolism, has been shown in multiple mouse models to enhance the cytotoxic function of CD8^+^ Tc1 cells, with its levels negatively correlated with tumor burden, indicating notable antitumor immune activity. In human studies, microbiota genes encoding formate-producing enzymes were enriched in ICI responders, further highlighting the clinical potential of formate as a novel immunoregulatory metabolite (71, 72).

Urocanic acid (UCA) is a newly identified microbiota-derived metabolite. Studies have shown that tyrosine kinase inhibitors (TKIs) markedly increase the abundance of Muribaculum in the gut microbiota, thereby promoting UCA production. Mechanistic investigations revealed that UCA binds to IκBα in tumor endothelial cells, thereby inhibiting the NF-κB–CXCL1 signaling pathway, reducing myeloid-derived suppressor cell (MDSC) infiltration, improving the immune microenvironment, and enhancing the efficacy of anti-PD-1 therapy. These findings suggest that UCA may serve as a key metabolic mediator of TKI–ICB combination therapy and holds clinical potential as an immunotherapy sensitizer (77).

Metabolomic analyses have identified gut microbiota–derived 4-hydroxyphenylacetic acid (4-HPA) as a pro-tumor metabolite that accumulates in colorectal cancer, especially in advanced disease, and is closely associated with an immunosuppressive tumor microenvironment. Mechanistically, 4-HPA activates JAK2/STAT3 signaling in CRC cells to induce CXCL3 expression, thereby recruiting PMN-MDSCs via CXCR2 and impairing CD8^+^ T cell effector function, ultimately promoting ICI resistance. Notably, inhibition of the shikimic acid pathway by chlorogenic acid (CGA) reduces 4-HPA production, restores antitumor immunity, and improves PD-1 blockade efficacy, highlighting 4-HPA as a key metabolic driver of immune evasion and a potential therapeutic target (78).

Recent studies identified L-selenomethionine (L-SeMet), a microbiota-derived metabolite, as a potent enhancer of CD8^+^ T cell function. L-SeMet boosts IFN-γ, TNF-α, granzyme B, and IL-2 secretion, amplifies T cell receptor (TCR) signaling via LCK-dependent phosphorylation, and strengthens antitumor cytotoxicity. In mouse CRC models, oral L-SeMet suppressed tumor growth and enhanced CD8^+^ T cell effector activity, highlighting its potential as a dietary selenium–based immunotherapy sensitizer (79).

#### 2.6.2 Immune-cell regulation by emerging metabolites

TMAO activates the NOD-like receptor family pyrin domain-containing 3 (NLRP3) to promote M1 polarization and enhance CD8^+^ function (149). Urolithin A improves mitochondrial fitness and restores NK and CD8^+^ cytotoxicity (69, 70). Inosine boosts T-cell activity through A2AR–cAMP–PKA signaling (150, 151) yet can stimulate Tregs and MDSCs (152). Formate triggers Nrf2 activation and IFN-γ release (68, 69), while arginase-driven arginine depletion suppresses T-cell responses (63–66). Together, these emerging metabolites bridge metabolic reprogramming and immune activation, shaping tumor immunity and the response to immune-checkpoint blockade.

## 3 Microbiota-derived metabolites in GI cancer immunotherapy

### 3.1 Gut microbiota diversity and the efficacy of PD-1/PD-L1 inhibitors

ICIs, particularly those targeting the PD-1/PD-L1 axis, have profoundly reshaped the therapeutic landscape of multiple cancers (153). However, their overall response rate remains limited, with challenges including resistance and immune-related adverse events. Increasing evidence suggests that gut microbiota diversity and community composition influence ICI efficacy, clinical studies and reviews consistently report an association between higher microbial diversity and improved clinical outcomes (11).In HCC, cohort findings remain inconsistent, responders have been associated with enrichment of *Lachnoclostridium, Lachnospiraceae, Veillonella*, and higher fecal UDCA, while retrospective studies also link *Akkermansia, Ruminococcaceae*, and Bifidobacterium to favorable prognosis. These heterogeneous results require validation in larger, standardized cohorts (154).

*Akkermansia muciniphila* (Akk), regarded as a “next-generation probiotic,” can significantly enhance the efficacy of PD-1/PD-L1 blockade by reshaping the immune ecosystem and boosting CD8^+^ T-cell function. Key mediators include the outer membrane protein Amuc_1100 and the protease Amuc_1434, which also regulate immune responses through modulation of SCFA and bile acid metabolism (155). In HCC cohorts, its abundance correlates with elevated UDCA/TUDCA levels, suggesting that Akk may improve ICI efficacy through bile acid metabolism. Beyond functional bacterial strains, dietary components can also shape the microbiota and metabolic profiles. For instance, Chinese yam polysaccharide (CYP) has been shown to synergize with PD-1 therapy in CRC mice by improving microbial diversity and reducing deoxyguanosine (dG) levels, thus preventing M2 polarization and reversing immunosuppression (156).

### 3.2 Regulatory effects of microbiota-derived metabolites on ICIs efficacy

Building on microbial diversity, microbiota-derived metabolites are regarded as functional mediators that directly account for differences in ICI efficacy. Distinct classes of metabolites can exert either positive or negative effects on immunotherapy through signaling pathways, metabolic reprogramming, and immune cell regulation.

As key metabolites of the gut microbiota, SCFAs have attracted increasing attention for their immunosensitizing effects in ICI therapy. Multiple preclinical studies suggest that SCFAs enhance antigen presentation and promote CD8^+^ T-cell effector functions, thereby augmenting the antitumor efficacy of PD-1/PD-L1 blockade (157, 158).

As key metabolites of the gut microbiota, SCFAs have recently gained significant attention for their immunosensitizing effects in ICI therapy. Multiple preclinical studies suggest that SCFAs can enhance antigen presentation and promote CD8^+^ T-cell effector functions, thereby augmenting the antitumor efficacy of PD-1/PD-L1 blockade. Among them, butyrate is particularly representative, as it enhances CD8^+^ T-cell activity through epigenetic mechanisms such as HDACs inhibition, generating a synergistic effect with PD-1 blockade (159, 160). In gastric cancer models, microbiota-derived butyrate downregulates PD-L1 and IL-10 expression in TAMs, thereby alleviating immunosuppression and reinforcing antitumor responses (161). Evidence at the bacterial strain level further supports this conclusion. For example, *Faecalibacterium prausnitzii* (EXL01) restores immune function after antibiotic-induced damage and enhances PD-1/PD-L1 efficacy (162). Similarly, the butyrate-producing Clostridium butyricum strengthens antitumor responses to PD-1 therapy in colorectal cancer models by suppressing IL-6–mediated immunosuppression. Notably, the clinically promising CBM588 strain has been shown to promote CD8^+^ T-cell infiltration and functional activation, thereby prolonging survival. However, under inflammatory conditions, CBM588 may also exacerbate inflammation, highlighting the need for context-specific application (163). These findings suggest that butyrate and its producing bacteria act not only as metabolite-level immunosensitizers but also hold translational potential. However, their effects remain context-dependent: while enhancing PD-1 blockade, SCFAs may attenuate CTLA-4 inhibition by promoting Treg expansion, underscoring the heterogeneity of their immunoregulatory roles across therapeutic settings (155, 164).

Moreover, the ability of SCFAs to potentiate PD-1 blockade efficacy is highly dependent on molecular and immunological context. Studies have shown that butyrate and propionate can induce the DNA damage response (DDR), upregulate antigen-presenting molecules (MHC-I) and chemokines, thereby enhancing CD8^+^ T-cell infiltration and effector function, ultimately improving the antitumor efficacy of PD-1 blockade (160). In microsatellite instability–high (MSI-H) colorectal cancer (CRC), tumors already exhibit a high mutational burden and abundant neoantigens, resulting in relatively high response rates to PD-1 blockade. In this context, SCFAs mainly act to further enhance effector T-cell function and sustain immune-inflammatory activity. In contrast, microsatellite-stable (MSS) CRC is typically resistant to PD-1 therapy. However, preclinical studies demonstrate that propionate, in synergy with *Bacteroides fragilis*, can reshape the immunosuppressive tumor microenvironment and markedly enhance PD-1 efficacy. This offers a new therapeutic entry point for MSS CRC, which has traditionally been unresponsive to ICIs (165, 166). Both clinical and mechanistic evidence are steadily accumulating: in solid tumor patients treated with PD-1 inhibitors, higher SCFA levels in blood or feces are often associated with longer PFS. Functional studies further reveal that metabolites such as isobutyric acid and butyrate can enhance CD8^+^ T-cell cytotoxic activity by modulating TCR signaling, thereby providing immunosensitizing support for ICIs at the metabolite level (159, 167).

Bile acids and their metabolites regulate the immune microenvironment through receptors such as FXR and TGR5, and are closely associated with the efficacy of ICIs. Multiple studies have suggested that secondary bile acid profiles correlate with PD-1 responsiveness. In HCC cohorts, responders exhibited relatively higher fecal levels of UDCA and TUDCA, which were associated with PD-L1 downregulation and enhanced CD8^+^ T-cell function (168). In preclinical models, UDCA worked with anti–PD-1 therapy to augment CD8^+^ T-cell effector functions (169). Mechanisms included carboxy terminus of Hsc70-interacting protein (CHIP)–mediated TGF-β degradation, Treg suppression, and EMT downregulation through the TGR5–Yes-associated protein (YAP) axis, thereby relieving the immunosuppressive TME. Other studies have shown that *Akkermansia muciniphila* can increase TUDCA levels in parallel with tumor PD-L1 downregulation, suggesting that a “bile acid–PD-1/PD-L1–T-cell” axis may represent a novel target for immunotherapy (170). More recently, Jin et al. reported that specific secondary bile acids antagonize the androgen receptor (AR), activate CD8^+^ T cells, and enhance anti–PD-1 efficacy, providing new molecular evidence for the role of bile acids in sensitizing tumors to immunotherapy (171).

In GI cancers, the IDO/TDO–Kyn–AhR axis has been well established as a key driver of immunosuppression. By activating AhR, Kyn induces Treg differentiation, suppresses CD8^+^ T-cell function, and promotes immune checkpoint upregulation, thereby attenuating the efficacy of immunotherapy (172). In colorectal and hepatocellular carcinoma models, blockade of Kyn synthesis or transport has been shown to restore T-cell function and act synergistically with PD-1 blockade (173, 174).

Purine metabolism likewise plays a pivotal role in immune evasion in GI cancers. Among its pathways, the adenosine–A2A receptor axis has been extensively demonstrated to suppress effector T-cell and NK-cell activity while promoting the accumulation of immunosuppressive cell populations, constituting a major factor underlying immunotherapy resistance in colorectal and gastric cancers (175). Blocking adenosine signaling can remodel the tumor microenvironment and enhance the efficacy of PD-1/PD-L1 blockade (176).

Indole derivatives have been shown to activate the AhR–IL-22 pathway to improve mucosal barrier homeostasis, while also alleviating Kyn–AhR–mediated T-cell dysfunction in multiple models, thereby promoting responsiveness to ICIs. Further studies revealed that IPA upregulates the TCF7 super-enhancer, expands PD-1^+^/TCF1^+^ stem-like CD8^+^ T cells, and enhances the efficacy of anti–PD-1 therapy (177). IAld, via the AhR–cAMP response element-binding protein (CREB) axis, reinforces effector programming and sensitizes tumors to PD-(L)1 therapy (178). Meanwhile, IAA exhibits a typical dose- and context-dependent pattern. At moderate levels, IAA promotes mucin sulfation and maintains barrier and immune homeostasis through the AhR–3′-phosphoadenosine 5′-phosphosulfate synthase 2 (Papss2)–solute carrier family 35 member B3 (Slc35b3) pathway. However, at high concentrations or within specific tumor microenvironments, IAA may excessively activate AhR, skewing toward immunosuppressive networks and attenuating CD8^+^ T-cell effector function, ultimately diminishing the efficacy of PD-1/PD-L1 blockade (179). The functional direction of IAA also varies across GI cancers: in colorectal cancer, it predominantly exerts barrier-protective and antiproliferative effects, whereas in pancreatic cancer, it may instead promote immune evasion (48, 172).

Inosine, a purine metabolite, has recently attracted attention for its immunosensitizing effects. Studies indicate that inosine, acting as a ligand of the A2A receptor, activates the cAMP–PKA signaling pathway to enhance CD8^+^ T-cell metabolic adaptability and effector function (141). In GI tumor models, exogenous inosine supplementation synergizes with PD-1 or CTLA-4 blockade to improve antitumor immunity (150), suggesting its potential translational value in optimizing immunotherapy efficacy.

TMAO has been shown in pancreatic cancer models to drive TAMs toward an immune-activating phenotype and to enhance the effector functions of intratumoral IFNγ^+^TNFα^+^ T cells. When combined with ICB, TMAO significantly reduces tumor burden and prolongs survival, an effect dependent on type I interferon (IFN-I) signaling (67).

Exercise-induced microbiota-derived metabolite formate has recently been identified as a potential sensitizer for ICIs. Mechanistic studies demonstrate that formate activates CD8^+^ T-cell effector functions via the nuclear factor erythroid 2–related factor 2 (Nrf2) signaling pathway, enhancing IFN-γ and granzyme B secretion, thereby amplifying the antitumor immune response to PD-1/PD-L1 blockade. Fecal microbiota transplantation and human cohort analyses further indicate that a high formate-producing microbiota is positively correlated with improved ICI responsiveness (71), providing new therapeutic avenues for combining exercise or dietary interventions with ICIs.

## 4 Clinical prospects of microbiota metabolites in GI cancer immunotherapy

### 4.1 Dietary interventions and metabolite modulation

Diet directly shapes the gut microbiota and its metabolites, influencing ICI efficacy through the “diet–microbiota–metabolite” axis. High-salt diets promote pro-inflammatory metabolites like skatole, driving immunosuppression, whereas indoles such as IPA and IAA improve the tumor immune microenvironment and T-cell function. Thus, dietary modulation of tryptophan metabolism can critically affect immunotherapy outcomes, underscoring the potential of diet as an adjunct strategy (180). Multiple studies have demonstrated that a high-fiber diet significantly elevates SCFA levels, thereby enhancing dendritic cell antigen presentation and effector T-cell function, ultimately improving responses to ICIs (181). High-fiber intake and Mediterranean diets are associated with improved ICI outcomes through enhanced microbial diversity and butyrate production, whereas antibiotics, PPIs, and corticosteroids reduce efficacy. These findings highlight diet–microbiota interactions as both predictive markers and modifiable strategies to optimize immunotherapy (181). The underlying mechanisms through which various dietary patterns enhance immune checkpoint inhibitor (ICI) efficacy are summarized in **Table 2**.

**Table 2:** Mechanisms by which different diets promote ICIs efficacy.

Dietary	Metabolite/microbiota changes	Major mechanisms	Impact on the efficacy of ICIs		Refs.
High-salt diet	↑ Skatole (3-methylindole)	Induces macrophage secretion of IL-1β and TNF-α	Weaken antitumor efficacy	(180)	
High-fiber diet	↑ SCFAs, ↑ probiotic diversity	Enhances DC antigen presentation; improves CD8^+^ T-cell function	Increases ICI response rate; effects vary across models	(181)	
Mediterranean diet	↑ Polyphenols, ↑ butyrate-producing bacteria	Increases microbiota diversity and SCFA levels	Presumed to improve immunotherapy response	(181)	
Adverse interventions (antibiotics, PPIs, glucocorticoids)	↓ Microbiota diversity	Disrupts microbiota–metabolite homeostasis	Weakens ICI efficacy	(181)	
Ketogenic diet/Choline supplementation	↑ TMAO-related metabolism	Regulates the TMAO–immune activation axis	Enhances immune activation	(183)	
Exercise/Intermittent fasting	↑ Butyrate-producing bacteria	Enhances SCFAs, increases immune sensitivity	Improves anti–PD-1 efficacy	(185, 186)	
Pectin	↑ Butyrate-producing bacteria, ↑ CD8^+^ infiltration	Enhances antigen presentation & T-cell effector function	Enhances anti–PD-1 efficacy	(187)	
Inulin	↑ *Lactobacillus, Roseburia, Akkermansia*;↑ SCFAs	Regulates the metabolic profile	Improves PD-1 blockade efficacy	(188)	
Ginseng polysaccharides	↑ Valerate, ↓ Kyn; ↑ *Lachnospiraceae*	Inhibits Tregs, promotes CD8^+^ function, ↓ MDSCs	Sensitizes anti–PD-1 efficacy	(190)	
LBP, anthocyanins, GLP, flavonoids	↑ SCFAs, ↑ L-Arg metabolism	Enhances immune response	Synergizes with ICIs	(189)	
Sea cucumber polysaccharides	↑ *Lactobacillus gasseri, Lactobacillus reuteri, Bifidobacterium pseudolongum;* ↑ I3C	↑ CD8^+^ function, ↓ Tregs, improves immune microenvironment	Synergizes with anti–PD-1 to inhibit tumors	(192)	
Huaier polysaccharides	↑ *Akkermansia, ↓ Parasutterella*; ↓ TCDCA	Inhibits M2 polarization, ↑ M1/M2 ratio, enhances pro-inflammatory cytokines	Inhibits HCC growth, improves immune microenvironment	(193)	
Ginger-derived exosome-like nanoparticles	↑ DHA enrichment, ↓ PD-L1 transcription	Regulates microbiota metabolism via miR-159a	Enhances anti–PD-L1 efficacy	(191)	

SCFAs, Short-chain fatty acids; IL, Interleukin; TMAO, Trimethylamine N-oxide; TNF-α, tumor necrosis factor-alpha; PD-1, Programmed cell death protein 1; PD-L1, Programmed death-ligand 1; M1/M2, M1 macrophages/M2 macrophages; CD8^+^/CD4^+^, CD8^+^/CD4^+^ T lymphocyte; HCC, Hepatocellular carcinoma; CRC, Colorectal Cancer; MDSCs, Myeloid-Derived Suppressor Cells; Treg, Regulatory T cell; LBP, Lycium barbarum polysaccharides; GLP, Ganoderma lucidum polysaccharides; TCDCA, taurochenodeoxycholic acid;I3C, indole-3-carboxylic acid; ICB, immune checkpoint blockade; DC, Dendritic cell; ICIs, immune checkpoint inhibitors; Kyn, kynurenine. DHA, Docosahexaenoic acid; ↑, increase; ↓, decrease.

However, a recent study across five murine tumor models indicated that simply increasing dietary fiber does not consistently potentiate ICI efficacy; rather, baseline dietary composition and non-fiber components exert stronger effects on gut microbiota composition, metabolite profiles, and tumor immunity (182). Building on this, other dietary patterns are gaining attention. For instance, ketogenic diets and choline supplementation may enhance immune activation via TMAO metabolism (183, 184), while exercise and intermittent fasting promote the abundance of butyrate-producing bacteria and increase immune sensitivity (185, 186). Dietary fibers and polyphenols enhance mucosal barrier function by driving beneficial indole metabolites and can potentiate immunotherapy through the “gut microbiota–metabolite” axis. In preclinical models, pectin boosts anti–PD-1 efficacy via butyrate-producing bacteria and CD8^+^ T-cell infiltration, while inulin increases probiotic abundance, SCFAs, and immune metabolites to improve PD-1 blockade (187, 188).

Similarly, components such as Lycium barbarum polysaccharides, anthocyanins, Ganoderma lucidum polysaccharides, flavonoids, and Inonotus hispidus have been reported to exert comparable effects, mainly by facilitating probiotic colonization and enhancing SCFA or L-arginine metabolism to improve antitumor immunity (189). Ginseng polysaccharides (GPS), in particular, have been found to increase valerate levels and decrease kynurenine, thereby suppressing Treg activity and promoting effector T-cell differentiation. In CRC models, GPS enhances CD8^+^ T-cell function and suppresses MDSCs by promoting *Lachnospiraceae* colonization and SCFA production. Clinical sample analyses further confirmed that *Lachnospiraceae* abundance correlates positively with CD8^+^ T cells and negatively with MDSCs, underscoring its potential as an immunotherapy sensitizer in CRC (190). Plant-derived nanoparticles (PNPs) show promise as immunotherapy sensitizers. Ginger-derived exosome-like nanoparticles (GELNs) reshape microbial metabolism via miR-159a, enriching DHA and downregulating PD-L1 to enhance anti–PD-L1 efficacy, with clinical data linking higher DHA to lower PD-L1 expression (191). Sea cucumber polysaccharides (SCPs) synergize with PD-1 blockade in CRC models by enhancing CD8^+^ T-cell function, reducing Tregs, and remodeling the microbiota to elevate indole-3-carboxylic acid. Fecal microbiota transplantation (FMT) confirmed this microbiota-dependent effect, highlighting SCPs as promising dietary adjuvants in immunotherapy (192). Huaier polysaccharides (HP) show strong antitumor effects in HCC models by remodeling bile acid metabolism, notably reducing TCDCA. This shift suppresses M2 macrophage polarization, increases the M1/M2 ratio, and boosts pro-inflammatory cytokines, thereby reversing immunosuppression (193).

Diet shapes the “microbiota–metabolite–immunity” axis, where balanced nutrition enhances antitumor immunity, while unhealthy patterns foster pro-inflammatory metabolites and weaken immunotherapy efficacy.

### 4.2 Prospects for probiotic applications

The butyrate-producing probiotic Clostridium butyricum (CBM588) enhances antitumor immunity by expanding IL-17A^+^ T-cell subsets and modulating gut microbiota (194). Preclinical and early clinical studies show that CBM588 combined with PD-1/PD-L1 or PD-1/CTLA-4 therapy improves response rates and PFS, underscoring the clinical potential of functional probiotics (195).

In colorectal cancer liver metastasis models, Kang’ai Jinfang (KAJF) reshapes the immune microenvironment by enriching SCFA-producing bacteria and elevating butyrate, which improves barrier function and suppresses M2 macrophages. KAJF also reduces MDSCs and enhances CD8^+^ T-cell infiltration, thereby inhibiting metastatic growth (196). In addition to butyrate-producing bacteria, other probiotics such as *Akkermansia muciniphila* and *Bifidobacterium* have also been shown to enhance PD-1/PD-L1 blockade efficacy by reshaping metabolic profiles and optimizing the tumor immune microenvironment. For example, *Bifidobacterium*-derived inosine can augment CD8^+^ T-cell effector functions in the context of PD-1/CTLA-4 blockade (197). Further studies revealed that Bifidobacterium activates the cyclic GMP–AMP synthase–stimulator of interferon genes (cGAS–STING) pathway, enhancing dendritic cell function and CD8^+^ T-cell responses, thereby improving ICB efficacy (143).

*Akkermansia muciniphila* provides another representative case. Its metabolite c-di-AMP activates the STING–type I interferon pathway, thereby strengthening antitumor immunity and responsiveness to immune checkpoint therapy (198). In metabolic dysfunction–associated fatty liver disease (MAFLD)–associated HCC models, *Akkermansia* abundance declines during disease progression. In contrast, supplementation significantly improves the local immune landscape. It restores intestinal barrier integrity, reduces serum lipopolysaccharide (LPS) and bile acid levels, alleviates hepatic inflammation, diminishes infiltration of monocytic MDSCs and M2 macrophages, and promotes CD4^+^/CD8^+^ T-cell recruitment and activation, thereby reversing immunosuppression (27). In addition, *Bifidobacterium* and *Lactobacillus* species can also produce and release c-di-AMP, which acts as a STING agonist to trigger cGAS–STING–IFN-β signaling in dendritic cells. This strengthens type 1 conventional dendritic cell (cDC1)–mediated cross-priming and expands tumor-specific CD8^+^ T cells, creating synergy with PD-1 blockade and improving antitumor efficacy (199–201). Specific Lactobacillus species enhance ICB efficacy via tryptophan metabolism, *L. johnsonii* and *L. gallinarum* modulate CD8^+^ T-cell stemness and Treg differentiation, while *L. reuteri* generates indole-3-aldehyde to activate AhR signaling and boost IFN-γ^+^ CD8^+^ T-cell function (177, 202).

*Faecalibaculum* has been reported to enhance immunotherapy efficacy through inosine production, a process that depends on ICB-induced disruption of the intestinal barrier, which permits systemic translocation of inosine and subsequent activation of antitumor T cells (203). Furthermore, engineered microbial strategies have demonstrated even greater translational potential. Xu et al. reported that engineered Lactobacillus reuteri (LR-S-CD/CpG@LNPs), when administered orally in combination with near-infrared irradiation, induced immunogenic cell death in CRC tissues and promoted dendritic cell maturation as well as CD8^+^ T-cell infiltration. Meanwhile, it enhanced I3A production via tryptophan metabolism to activate AhR signaling, thereby markedly strengthening antitumor immunity (204). Similarly, oral delivery of the oncolytic magnetotactic bacterium (MSR-CPT/APP@LPs) has been shown to not only induce immunogenic cell death and activate the cGAS–STING pathway within tumors, but also to reprogram metabolism and optimize the gut microbiota–metabolite axis. This is characterized by elevated SCFA levels that enhance effector T-cell function, reduced kynurenine pathway metabolites that alleviate Treg induction, and decreased glutamine consumption that suppresses tumor energy metabolism (205). The mechanisms by which different bacterial strains promote ICI responsiveness are summarized in **Table 3**.

**Table 3:** Mechanisms by which different bacterial strains promote ICIs efficacy.

Strains	Metabolites/mechanisms	Major immune functions	Impact on the efficacy of ICIs	Refs.
*Clostridium butyricum (CBM588)*	↑ SCFAs (butyrate)	Promotes the expansion of IL-17A^+^ γδT cells and CD4^+^ T cells; suppresses IL-6/IL-10 production; improves intestinal barrier integrity; inhibits M2 polarization and reduces MDSCs.	Enhances the efficacy of PD-1/PD-L1 or PD-1/CTLA-4 therapy, improving response rate and PFS.	(194, 195)
KAJF enriches SCFA-producing bacteria	↑ butyrate	Inhibits M2 polarization and decreases IL-10/TGF-β; reduces MDSCs; increases CD8^+^ T-cell infiltration.	Significantly inhibits CRC liver metastasis	(196)
*Bifidobacterium*	↑ Inosine; activates cGAS–STING	Enhances CD8^+^ T-cell function; promotes DC activation.	Synergizes with PD-1/CTLA-4 blockade to improve response rate	(143, 197)
*Akkermansia muciniphila (Akk)*	c-di-AMP → STING–IFN-I axis; regulation of SCFAs and bile acids	Repairs barrier; reduces MDSCs and M2; ↑ CD4^+^/CD8^+^ recruitment; activates antitumor immunity.	Improves PD-1 efficacy in MAFLD-HCC and CRC.	(27, 198)
*Genus Lactobacillus (L. johnsonii, L. gallinarum, L. reuteri)*	Regulates tryptophan metabolism → ↑ IAld; produces c-di-AMP.	Regulates CD8^+^ stemness and Treg differentiation; IAld–AhR → ↑ IFN-γ^+^ CD8^+^; c-di-AMP → cGAS–STING activation.	Enhances PD-1/ICB efficacy	(177, 199, 202)
*Faecalibaculum*	↑ Inosine (dependent on ICB-induced barrier disruption)	Activates antitumor T cells	Sensitizes ICIs, but depends on specific barrier conditions.	(203)
Engineered *Lactobacillus reuteri* (LR-S-CD/CpG@LNPs)	↑ IAld (I3A); activates AhR signaling	Induces immunogenic cell death; ↑ DC maturation & CD8^+^ infiltration.	Induces immunogenic cell death; ↑ DC maturation & CD8^+^ infiltration.	(204)
Oncolytic magnetotactic bacteria (MSR-CPT/APP@LPs)	Metabolic reprogramming: ↑ SCFAs, ↓ Kyn, ↓ glutamine consumption.	Activates cGAS–STING; ↑ CD8^+^ function; alleviates immunosuppression.	Inhibits CRC growth and synergizes with PD-1.	(205)

SCFAs, Short-chain fatty acids; IL, Interleukin; MDSCs, Myeloid-Derived Suppressor Cells; PD-1, Programmed cell death protein 1; PD-L1, Programmed death-ligand 1; CTLA-4, Cytotoxic T-Lymphocyte Antigen-4; PFS, Progression-free survival; KAJF, A traditional Chinese medicine formula; M1/M2, M1 macrophages/M2 macrophages; TGF-β, Transforming Growth Factor-beta; CD8^+^/CD4^+^, CD8^+^/CD4^+^ T lymphocyte; MAFLD, Metabolic dysfunction-associated fatty liver disease; HCC, Hepatocellular carcinoma; CRC, Colorectal Cancer; IAld, Indole-3-aldehyde; c-di-AMP, cyclic di-adenosine monophosphate; Treg, Regulatory T cell; AhR, Aryl hydrocarbon Receptor; IFN-γ^+^, interferon-gamma positive; cGAS, cyclic GMP–AMP synthase; ICB, immune checkpoint blockade; DC, Dendritic cell; ICIs, immune checkpoint inhibitors; Kyn, kynurenine; ↑, increase; ↓, decrease; γδT cells, gamma-delta T cells; STING, stimulator of interferon genes.

These findings suggest that microbial therapies exert dual effects—activating immunity and relieving immunosuppression—through the microbiota–metabolite axis, highlighting the pivotal role of metabolites in reshaping the TME and enhancing immunotherapy efficacy.

## 5 Immune–organoid co-culture systems for microbiota–immune interactions

### 5.1 Limitations of legacy models

Despite the important contributions of conventional *in vitro* systems and mouse models to our understanding of gut microbiota–immune crosstalk, both approaches have notable limitations. *In vitro* platforms often lack a complete immune compartment or must rely on peripheral immune cells of heterogeneous origin, and it is technically challenging to maintain steady-state coexistence of an oxygen-requiring epithelium with strict anaerobic commensals within the same system (206). Mouse models, in turn, differ substantially from humans in anatomy, physiology, immune composition, and microbiome structure, which limits the clinical translatability of their findings. Moreover, widely used cancer cell lines are closer to a small-intestinal phenotype and therefore fail to recapitulate the physiological immune responses of colonic epithelium (207). Traditional three-dimensional intestinal organoids can support replication of enteric viruses, but because they inherently lack immune cell components, they cannot reproduce the complex dynamics of epithelial and immune interactions and inflammatory responses. The recently proposed MaugOs (macrophage-augmented gut organoids) address this gap by integrating human macrophages, enabling parallel analysis of viral infection, immune signaling, and inflammatory readouts on a single platform and thereby improving immunophysiological relevance (208). Comprehensive reviews likewise emphasize that organoids can closely reproduce the structure and function of native intestinal epithelium and have become powerful tools for studying TME interactions; however, their capacity to model systemic immune crosstalk and dynamic shifts in the microbiota remains limited (209).

### 5.2 Immune–organoid co-culture systems

With advances in three-dimensional organoid culture and strategies for integrating immune cells, immune–organoid co-culture systems are emerging as more physiologically relevant experimental platforms. In these systems, intestinal organoids derived from pluripotent or adult stem cells are co-cultured with dendritic cells, macrophages, or T lymphocytes to reconstruct the epithelial–immune interface under controlled conditions, while also sustaining long-term co-culture with strict anaerobic commensals. This configuration more accurately models the human gut immune microenvironment. For example, Recaldin and colleagues established human intestinal immuno-organoids (IIOs) by integrating autologous tissue-resident memory T cells (TRM) into epithelial organoids, thereby creating a self-organizing immune compartment that recapitulates intraepithelial T-cell migration, adhesion, and cytokine signaling (210). In parallel, the MaugOs (Macrophage-augmented Intestinal Organoids) platform demonstrates the functional advantages of immune-enhanced organoids by enabling concurrent analysis of viral infection, epithelial interferon-mediated antiviral pathways, and macrophage-driven inflammatory responses, and by distinguishing virus-specific immune profiles (208).Reviews centered on the gut microbiota–tumor microenvironment axis further emphasize that, although organoids capture tumor heterogeneity and architecture, they remain limited in reproducing global immune crosstalk. Consequently, incorporating immune cells into organoid systems to build immune–organoid co-cultures has become a key direction for improving physiological relevance and translational utility (209).

These systems also offer new strategies to dissect how gut microbial metabolites regulate the epithelial barrier and immune cell differentiation. In the GuMI-APC (Gut–Microbe Interface with Antigen-Presenting Cells) platform, the strict anaerobic commensal *Faecalibacterium prausnitzii* induces upregulation of epithelial genes such as TLR1 and interferon alpha 1 (IFNA1), while CD4^+^ T-cell–mediated feedback suppresses transcription of inflammation-related genes. These findings indicate that the composition and activation state of immune cells can rewire microbe–epithelium signaling and the associated cytokine networks (211). Further studies highlight SCFAs as the most well-substantiated beneficial metabolites. However, conventional *in vitro* experiments are limited by physiological constraints and absorption kinetics. Immune-competent organoid co-cultures and multi-organ microphysiological systems, including gut–liver and gut–liver–brain chips, are therefore required to resolve absorption, distribution, metabolism, and excretion and to localize sites of action in the host (212). Within the MaugOs model, acetate markedly suppresses inflammation triggered by LPS and, through inhibition of NLRP3 inflammasome, coordinates inflammatory and stress pathways across macrophages and epithelium. This reveals core mechanisms at the interface of metabolites and inflammation (208, 213). In both human and mouse systems, butyrate inhibits epithelial HDAC3 and, via the HDAC3–sprouty RTK signaling antagonist 2 (SPRY2) pathway, restricts tuft-cell differentiation, which in turn dampens the IL-13–tuft-cell–ILC2 axis and downregulates type-2 immunity (214). Type-2 responses in tumors often promote M2-like macrophage polarization, matrix remodeling, and immunosuppression, which correlate with poor prognosis and reduced sensitivity to ICB. Consequently, attenuation of type-2 immunity through the butyrate–HDAC3 pathway may alleviate such immunosuppression, although the net effect is context dependent and should be validated in immune–organoid co-culture platforms (215).

A recent study reported the PD-IO platform, which integrates patient-derived epithelial organoids, autologous T cells, and myeloid-derived suppressor cell (MDSC)–conditioned medium, with optional anti-PD-1 treatment using pembrolizumab, to reconstruct tumor–immune interactions and ICI response or resistance *in vitro*. This platform distinguished MSI from MSS responses and, using caspase-3/7 and granzyme B as functional readouts, identified regenerating family member 4 (REG4) and mucin family members such as MUC1 and MUC13 as markers associated with ICI resistance; targeted knockout restored sensitivity to PD-1 blockade (216).Taken together with the mechanistic insights from the butyrate–HDAC3–SPRY2 axis, these observations support evaluating butyrate-producing microbes or butyrate itself in combination with ICI within immune–organoid co-culture systems to accelerate personalized translation. Looking ahead, immune–organoid co-cultures are poised to become important preclinical platforms for testing metabolite-based immunologic adjuvants, probiotic interventions, and ICB combination strategies. Incorporating such systems into studies of gut microbiota–immune crosstalk will not only clarify metabolite-mediated mechanisms of immune regulation but also advance microbiota-informed precision immunotherapy toward clinical application.

## 6 Challenges and perspectives

A growing body of evidence demonstrates that gut microbiota-derived metabolites play pivotal roles in immune regulation and therapeutic responses in GI cancers. SCFAs, bile acids, tryptophan derivatives, as well as emerging metabolites such as purines, polyphenols, and vitamins, can influence antitumor immunity through mechanisms including metabolic reprogramming, epigenetic modification, and immune cell regulation. These metabolites may enhance antitumor immunity and improve ICI efficacy, but under certain conditions can also promote immunosuppression and resistance, exhibiting a characteristic “double-edged sword” effect. Thus, metabolite–immune interactions are not only central to understanding GI cancer pathogenesis but also provide new opportunities for optimizing immunotherapy strategies.

Nevertheless, current research faces multiple challenges. Metabolite effects are strongly influenced by endogenous factors such as concentration, tumor microenvironment, and interindividual variability, leading to marked heterogeneity across studies. This complexity also raises safety concerns regarding exogenous metabolite supplementation, which may trigger immune dysregulation or hepatotoxicity. Moreover, the gut microbiota–metabolite–immune axis itself is highly complex and readily influenced by diet, medications, and comorbidities, making causal relationships difficult to fully delineate. Most existing studies rely on animal models or small-scale clinical cohorts, often focusing on single metabolites, and their reproducibility and generalizability across cancer types remain uncertain. Additionally, the lack of standardized protocols for sample collection, detection techniques, and analytical methods hampers cross-study comparisons and limits the translational utility of metabolites as clinical biomarkers.

Future research should aim to elucidate causal relationships between metabolites and immune function at the mechanistic level, leveraging artificial intelligence and systems biology tools to map microbiota–metabolite–immune networks, and validating findings through humanized mouse models, organoids, and multi-omics integration. In particular, immune-competent organoid co-culture systems can be employed to precisely localize the sites of metabolite action, reconstruct physiological gradients, quantify exposure–response relationships, and pre-evaluate, *in vitro*, the feasibility and efficacy of metabolite- or microbe-based immunotherapeutic combination strategies. Combining dietary interventions, probiotics, engineered bacteria, and metabolite supplementation may offer multidimensional strategies to improve the immune microenvironment and potentiate ICI efficacy. Furthermore, developing predictive and stratification models based on metabolite signatures could enable dynamic monitoring and precise assessment of immunotherapy efficacy and toxicity. Large-scale, multicenter clinical studies across cancer types will be essential to validate key metabolites and microbial factors for clinical translation. With continued advances in mechanistic insights and translational applications, microbiota-derived metabolites are poised to serve as a critical bridge between host immunity and personalized therapy, opening new avenues for precision immunotherapy in GI cancers.
